# Role of Sphingosine-1-Phosphate in Mast Cell Functions and Asthma and Its Regulation by Non-Coding RNA

**DOI:** 10.3389/fimmu.2017.00587

**Published:** 2017-05-22

**Authors:** Rohit Saluja, Ashok Kumar, Manju Jain, Sudhir K. Goel, Aklank Jain

**Affiliations:** ^1^Department of Biochemistry, All India Institute of Medical Sciences, Bhopal, India; ^2^Centre for Biochemistry and Microbial Sciences, Central University of Punjab, Bathinda, India; ^3^Centre for Animal Sciences, Central University of Punjab, Bathinda, India

**Keywords:** sphingosine-1-phosphate, sphingosine kinases, sphingosine-1-phosphate receptor, mast cells, asthma, non-coding RNA

## Abstract

Sphingolipid metabolites are emerging as important signaling molecules in allergic diseases specifically asthma. One of the sphingolipid metabolite, sphingosine-1-phosphate (S1P), is involved in cell differentiation, proliferation, survival, migration, and angiogenesis. In the allergic diseases, alteration of S1P levels influences the differentiation and responsiveness of mast cells (MCs). S1P is synthesized by two sphingosine kinases (SphKs), sphingosine kinase 1, and sphingosine kinase 2. Engagement of IgE to the FcεRI receptor induces the activation of both the SphKs and generates S1P. Furthermore, SphKs are also essential to FcεRI-mediated MC activation. Activated MCs export S1P into the extracellular space and causes inflammatory response and tissue remodeling. S1P signaling has dual role in allergic responses. Activation of SphKs and secretion of S1P are required for MC activation; however, S1P signaling plays a vital role in the recovery from anaphylaxis. Several non-coding RNAs have been shown to play a crucial role in controlling the MC-associated inflammatory and allergic responses. Thus, S1P signaling pathway and its regulation by non-coding RNA could be explored as an exciting potential therapeutic target for asthma and other MC-associated diseases.

## Introduction

Mast cells (MCs) are best known to trigger IgE-dependent/independent allergic diseases. They also play a significant role in providing immunity to host in response to various infections (1). MCs have enough storage of bioactive molecules and mediators to perform innate and adaptive immune responses. However, the same bioactive molecules may damage the surrounding tissues and cause inflammation. MCs can be activated by diverse stimuli including allergens, pollens, and toxins and may release a spectrum of molecules, including preformed mediators (such as histamine, proteases, and other enzymes). They are accountable for numerous symptoms of allergic reactions including edema and enhanced vascular permeability (2). MCs release variety of cytokines (e.g., IL-4, IL-5, and IL-13) and chemokines that are responsible for recruitment and maturation of different immune cells. In addition, MCs synthesize and release variety of lipid mediators such as prostaglandins, leukotrienes, platelet-activating factor, and sphingosine-1-phosphate (S1P) (3). S1P mediates its actions both by acting as a ligand for five S1P receptors and also an intracellular second messenger (4). It is also known to play an important role in numerous pathophysiological responses including allergic reactions such as asthma and chronic obstructive pulmonary disease (COPD).

## Biosynthesis and Metabolism of S1P

**Figure 1:**
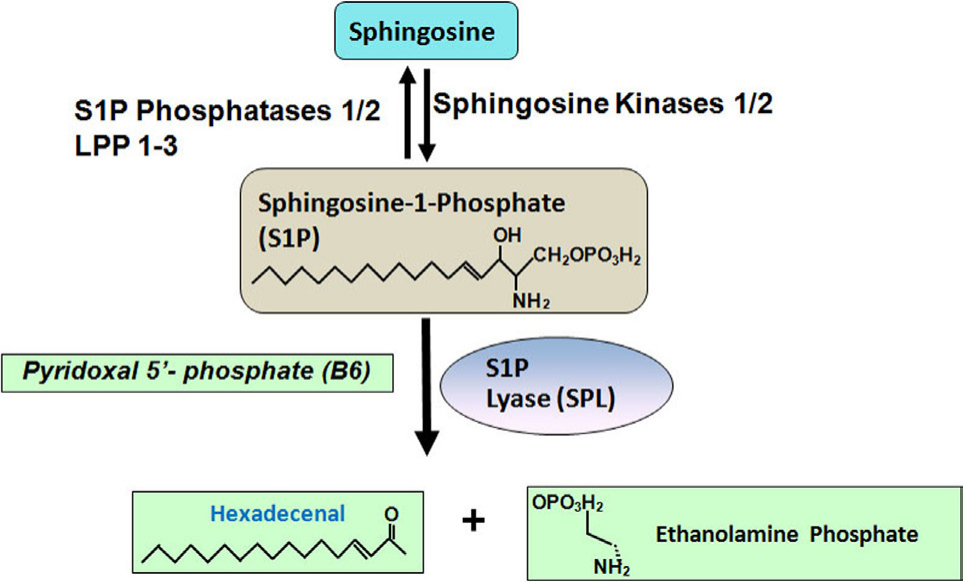
**Sphingosine-1-phosphate (S1P) is generated from sphingosine, which is catalyzed by sphingosine kinases 1/2**. S1P can be dephosphorylated by two S1P specific phosphatases, such as S1P phosphatase 1/2 and three non-specific lipid phosphatases, such as LPP 1–3. S1P can also be degraded into hexadecenal and ethanolamine phosphate by S1P lyase.

Two highly homologous sphingosine kinases (SphKs), known as sphingosine kinase 1 (SphK1) and sphingosine kinase 2 (SphK2) catalyze the synthesis of S1P from its precursor sphingosine (Figure 1) (4). SphK1 is mainly localized in the cytosol. Activation of SphK1 is regulated by many factors such as its intracellular localization, epigenetic, or posttranslational modification. SphK1 can be activated by a wide variety of growth factors, including epidermal growth factor, platelet-derived growth factor (PDGF), vascular endothelial growth factor, hepatocyte growth factor, and TNF-α (5). Unlike SphK1, SphK2 is localized in the nucleus and mitochondria-associated outer membrane (6, 7).

Circulating and tissue S1P levels are also regulated by its catabolism. There are six enzymes known to catabolize S1P. Two endoplasmic reticulum-bound, S1P-specific phosphatases, namely, S1P phosphatase-1 and -2 dephosphorylate S1P back to sphingosine. Three lipid phosphate phosphatases-1, -2 and -3 (LPP1–3) dephosphorylate a broad range of lipid phosphate substrates including S1P (8, 9). LPPs are located on the plasma membrane. Additionally, S1P lyase irreversibly degrades S1P to ethanolamine phosphate and *trans*-2-hexadecenal (10).

Till date, five S1P receptors (S1PR1–5) that bind to S1P have been identified in vertebrates (11). In mammalian cells, S1P receptors are ubiquitous, but their expression patterns vary among the different tissues. After coupling to G-proteins, these receptors either activate or inhibit downstream signaling pathways, including c-Jun N-terminal kinase, extracellular signal-regulated kinase, phosphatidylinositol 3-kinase, phospholipase C, phospholipase D, STAT3, Rho, Rac, and cyclic AMP (4). By activating these receptors, S1P regulates diverse biological processes including vascular development, angiogenesis, and immunity.

## Role of S1P in MC Activation

### FcεRI Induces S1P Generation and Its Export

**Figure 2:**
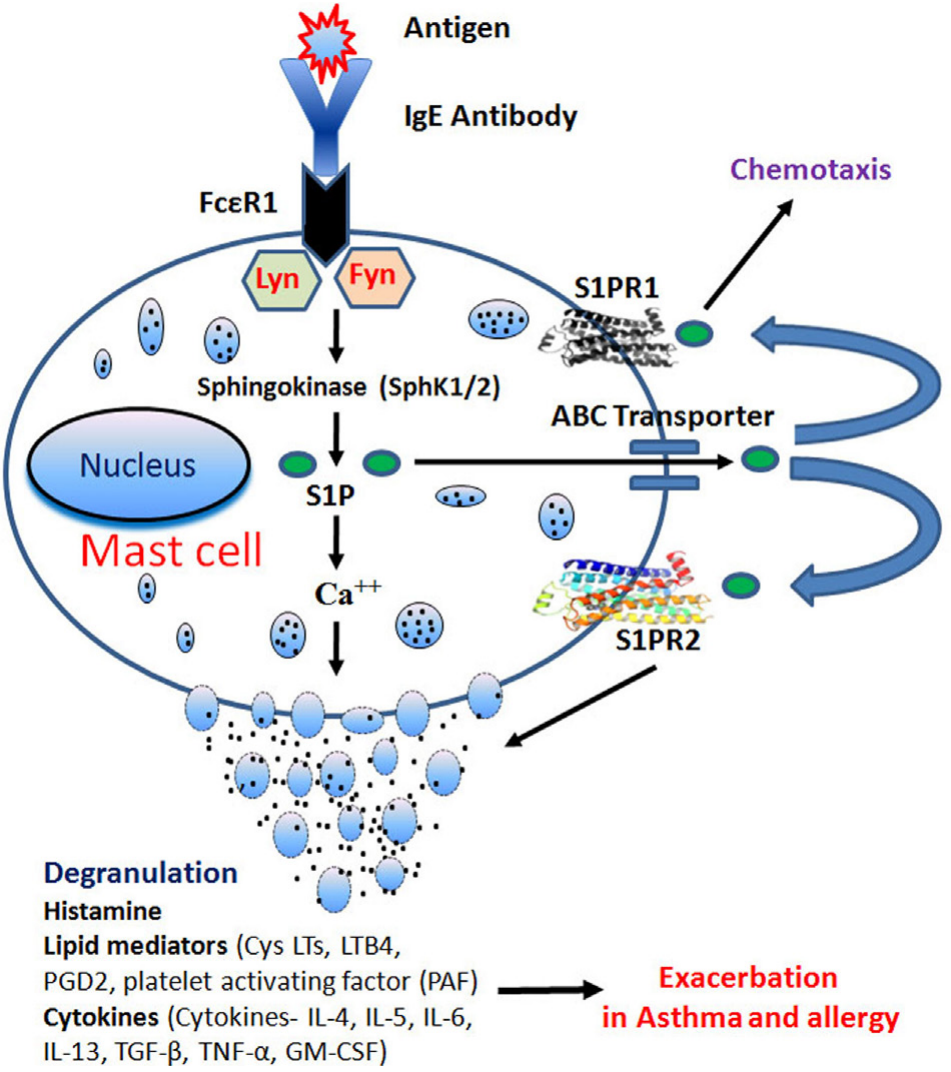
**IgE receptor crosslinking generates intracellular sphingosine-1-phosphate (S1P) through activation of sphingosine kinases (SphK1/2) in Lyn- and Fyn-dependent pathways**. S1P generation induces calcium mobilization leading to mast cells (MCs) degranulation. After degranulation, MCs release histamine, lipid mediators, and cytokines that play an important role in allergy and inflammation. S1P is also secreted through ATP-binding cassette (ABC) transporters and activates S1PR1 and S1PR2, present on MCs leading to chemotaxis and degranulation, respectively.

Antigen-induced aggregation of IgE bound to FcεRI on MCs activates both the SphKs with a consequent increase in intracellular S1P levels (12). Activation of SphK1 requires its translocation to the plasma membrane. Indeed, in bone marrow-derived mast cells (BMMCs), within minutes of FcεRI ligation, SphK1 is translocated to plasma membrane. Lyn, a src kinase is required for the early phase of SphK activation in MCs and promotes the recruitment of SphK1 to FcεRI. Lyn-deficient MCs show a delay in the activation of SphK by IgE/Ag (13). Likewise, Fyn, another src kinase, is also required for the activation of SphK1 and SphK2 in MCs. Furthermore, Fyn kinase interacts with SphK1 and SphK2 proteins, and Fyn-deficiency causes a complete ablation of SphK activation (Figure 2) (13, 14). IgE/Ag-induced activation of SphK1 occurs through Grb2-associated binder 2 (Gab2)-mediated pathway, whereas SphK2 is activated in a Gab2-independent manner (14). FcεRI-mediated activation of SphKs generates intracellular S1P, which is secreted into the extracellular space (14, 15). MCs can release copious amount of S1P upon agonist stimulation, which may be an important paracrine component of MCs (16). The ATP-binding cassette (ABC) superfamily of transporters has been shown to involve in the transport of S1P (17, 18). ABCC1, one of the ABC members, has been implicated in the FcεRI-stimulated export of S1P from RBL-2H3 and human LAD2 MCs (18).

### Role of SphKs in MC Activation

Upon engagement of FcεRI receptor, both the isoforms of SphKs get activated in MCs (19, 20). However, SphK1 and SphK2 might have some redundant functions in MCs, and their relative importance depends on the origin of MCs. Although, SphK2 is the major producer of S1P in mouse BMMCs (21), however, MC degranulation is not affected in SphK2-deficient BMMCs from adult mice. On the contrary, MCs-derived from liver progenitors of SphK2-deficient embryos consistently show a reduction in the degranulation. Surprisingly, MCs derived from liver progenitors of SphK1-deficient embryos exhibit no defect in degranulation (21). Furthermore, peritoneal-derived MCs (PDMCs) from either SphK1- or SphK2-deficient adult mice demonstrate impaired degranulation responses (19).

Similar discrepancy regarding the role of SphK1 or SphK2 in degranulation has been noted with human MCs. In CD34^+^ progenitors-derived human MCs (14) and in cord blood-derived human MCs (22), expression of both the SphK isoforms, SphK1 and SphK2, have been observed. Silencing of SphK1 by RNAi in the human MC line LAD2 shows that SphK1 is involved in degranulation (22). However, knockdown of SphK2 in LAD2 does not affect degranulation induced by Ag, ionomycin, or S1P but reduces the release of TNF-α and IL-6 (22). Furthermore, silencing of SphK1 or SphK2 using lentiviral-based short hairpin RNA reveals that SphK2 is required for degranulation, cytokine and leukotriene production, and calcium mobilization in murine MCs (19). In contrast, human MC response requires SphK1, which is more robustly expressed in human MCs (19). Nonetheless, all the studies carried out by pharmacological inhibitors, RNAi approaches, or genetic deletion models suggest a critical role of SphKs in MC function and point out that two isoforms may have some redundancy (20).

Calcium influx is a crucial process for FcεRI-mediated MC degranulation and cytokine generation (23). Fetal liver-derived MCs from SphK2-knockout mice show deficient calcium influx, cytokine and leukotriene release, and PKC activation, resulting into impaired MC degranulation (21). Role of SphK2 in calcium influx has also been confirmed in BMMCs and PDMCs (19). Addition of S1P to BMMCs does not show any effect on FcεRI-induced calcium mobilization, suggesting that S1P-induced calcium mobilization occurs through an S1P receptor-independent pathway. It has been hypothesized that sphingosine and S1P have opposing functions in regulating the opening of calcium channels in MCs (21). According to this notion, sphingosine inhibits MC degranulation, generation of leukotrienes and TNF-α; and S1P reverses these effects (15). MCs express low levels of IL-33, which is further induced upon IgE-mediated activation through a calcium-dependent mechanism. Inhibition of Sphk activity or short hairpin-mediated SphK1-silencing blocks calcium flux and decreases IL-33 expression induced by IgE/Ag activation (24).

### Role of S1P Receptors in MC Degranulation

Mast cells have been shown to express S1PR1, S1PR2, and S1PR4 but not S1PR3 and S1PR5 (20, 25). FcεRI ligation-mediated activation of SphKs induces a ligand-dependent trans-activation of S1PR1 and S1PR2, resulting in enhanced functions (25). S1PR1 is involved in the migration of MCs toward low concentrations of antigen whereas S1PR2 participates in FcεRI-induced degranulation (25, 26). It has been proposed that S1PR1 participates in the recruitment of MCs to their site of action, while S1PR2 deters migration and contributes to degranulation once MCs reach at the site of action. This also reinforces that SphK activation, S1P generation, and S1PR1–2 trans-activation are necessary for MC chemotaxis and degranulation (20).

Knockdown of *S1PR2* by RNA silencing or its genetic deletion in BMMCs significantly reduces FcεRI-induced degranulation (25). In a mouse model of anaphylaxis, S1PR2 antagonist JTE-013 markedly attenuates the severe hypothermia and reduces serum histamine levels (26). Further, the severity of anaphylaxis in S1PR2-deficient mice was significantly less as compared with their wild-type counterparts (26). Vaccinia virus causes degranulation of skin MCs in an S1P–S1PR-dependent pathway that leads to antimicrobial peptide discharge and virus inactivation (27). In contrast, a study has reported that S1PR2 is dispensable for the degranulation of mouse connective tissue type MCs, and it is not involved in the onset of IgE/Ag-mediated anaphylaxis (28).

### S1P-Mediated Regulation of MC Function

Circulatory S1P levels have been correlated with the degranulation capability of MCs. SphK1 deficiency in mice reduces plasma S1P levels, whereas SphK2 deficiency increases its levels (21, 29). Elevation of circulating S1P in SphK2-deficient mice may be due to a compensatory elevation of SphK1 activity in RBCs, which is the main source of plasma S1P. There is discordance between *in vitro* and *in vivo* data of the degranulation response in SphK2-deficient MCs. SphK2-deficient MCs exhibit defective degranulation *in vitro* in response to IgE receptor crosslinking. However, passive systemic anaphylactic mice model reveals a defect in the anaphylactic response in SphK1-knockout mice but not in SphK2-knockout mice (21). In contrast, SphK1-deficient MCs show normal degranulation *in vitro*; however, decreased histamine release upon a systemic challenge has been noted in SphK1-knockout mice. Further, SphK1- or SphK2-deficient mice show a strong association between the plasma S1P concentration and circulating histamine. These studies suggest that elevated levels of S1P in SphK2-deficient mice may lead to normal MC functions (21). It has been suggested that in systemic anaphylaxis, changes in the levels of circulating S1P may influence the responsiveness of the MCs and thus may affect the sensitivity and/or severity in *in vivo* response (20). Further, elevation of circulating S1P levels in C57BL/6 mice by an S1P lyase inhibitor, 2-acetyl-4-tetrahydroxybutyl imidazole, results in enhanced MC response in the mice following a systemic challenge (30). Furthermore, genetic background of experimental mice also influences MC response. Mice strain 129Sv, which has higher levels of circulating S1P, exhibit increased Th2-cell responses and anaphylaxis, compared to C57BL/6 mice that have low Th2-cell responses (31, 32). Moreover, chronic treatment of S1P during murine BMMC differentiation induces genetic changes leading to hyperresponsive MCs. In contrast, mice, lacking both the isoforms of SphKs, having undetectable levels of circulating S1P, show normal MC response to an anaphylactic inducer when challenged with strong stimuli (33).

## The Role of S1P Signaling in Asthma

Asthma may occur due to allergic/non-allergic reactions based on production of IgE antibodies to common environmental allergens (34). MCs, T lymphocytes, and eosinophils together with the resident airway cells interact with one another to perpetuate airway inflammation, leading to AHR and remodeling (35). A subset of human lung MCs, expressing CD88 receptor get activated by C5a ligand (36). Various autocoid mediators are released from activated MCs that may induce bronchoconstriction, vascular permeability, recruitment of inflammatory cells, mucous secretion, and tissue edema (35). The correlation between inflammatory cells and airway smooth muscle (ASM) cells plays a fundamental role in the pathophysiology of asthma. MCs from asthmatic patients have been shown to localize in the intercellular spaces of bronchial epithelium, ASM cells, and airway mucous glands. Thus, the disordered airway physiology and wall remodeling features of asthma are consequences of inflammation and bronchial hyperresponsiveness. In response to allergen challenge, activated MCs release inflammatory cytokines and other mediators that affect bronchial epithelial functions. Furthermore, it has been suggested that the density of MCs within the mucous gland influences the degree of mucus obstruction in the airway. MC-derived inflammatory cytokines also contribute to several features of asthma (35).

Extracellular S1P not only affects eosinophil infiltration of the airway wall but also stimulate the contractions of ASM by inducing calcium mobilization and augmenting the production of inflammatory cytokines. Moreover, S1P disrupts the epithelial cell barrier integrity (tight junctions) in the respiratory system (37–39). In peripheral airways, activation of cholinergic receptors, particularly muscarinic receptor, is associated with asthma and COPD. In mice, SphK1 has been shown to localize in the ASM of the peripheral airways. Furthermore, inhibition of SphK enzyme activity reduces muscarine-induced peripheral airway constriction, implying that the S1P signaling pathway contributes to cholinergic constriction of peripheral airways (40). Elevated levels of S1P have been observed in bronchoalveolar lavage (BAL) fluid of asthmatic patients, suggesting an important role of S1P in MC-dependent inflammatory responses in allergic reactions (38). S1P also acts as a chemotactic agent for eosinophils through RANTES and CCR3, further implying an involvement of S1P in the pathophysiology of asthma (41). Moreover, eosinophil numbers correlate with S1P levels in BAL of asthmatic patients (38). Antigen-stimulated MCs release S1P into interstitium, which may significantly modulate inflammatory processes. Interestingly, treatment with SphK inhibitor improves immune responses in mouse model of asthma (42).

In ovalbumin (OVA)-sensitized mice, S1P/SphK pathway triggers airway hyperresponsiveness (43). Furthermore, intratracheal instillation of FTY720 into mice reduces antigen-induced airway inflammation and hyperresponsiveness (44). On contrary, in asthmatic mouse model, intranasal S1P treatment results in exacerbation of antigen-induced airway inflammation and increased sensitivity to methacholine (45). Disodium cromoglycate (DSCG) treatment inhibits S1P-induced asthma-like features in mice. DSCG decreases the recruitment of MCs and B cells in the lung and reduces the levels of serum IgE, prostaglandin D_2_, mucus production, and IL-13 (46). S1P-stimulated ASM cells release elevated levels of IL-6 and RANTES (47). Furthermore, S1P administration to BALB/c mice increases ASM sensitivity, mucus production, and release of IgE, PGD_2_, IL-13 and IL-4, and increased recruitment of MCs to the lung (41). In MC-deficient mice, S1P does not induce bronchial hyperresponsiveness, suggesting that MCs are essential for S1P-induced bronchial hyperreactivity. Furthermore, S1P induces lung inflammation and ASM hyperreactivity in an IgE-dependent manner (41). Interestingly, in nude mice, S1P does not induce bronchial AHR, IgE release, and pulmonary infiltration of MCs. These data suggest that S1P-induced ASM hyperreactivity is T-cell dependent. Reconstitution of CD4^+^ T cells, isolated from S1P-treated mice to naïve (untreated) mice, recapitulated the ASM reactivity (41). Pre-exposure of S1P enhances methacholine-induced contraction of isolated ASM by Ca^2+^ sensitization *via* inactivation of RhoA-mediated myosin phosphatase (39). In a murine model of chronic asthma, SK1-I, a specific inhibitor of SphK1, significantly reduces OVA-induced AHR to methacholine. SK1-I also decreases eosinophil numbers and levels of different cytokines such as IL-4, IL-5, IL-6, IL-13, IFN-γ, and TNF-α and the chemokines, such as eotaxin, and CCL2 in BAL fluid (48).

## Non-Coding RNA in MC Activation and Asthma

Recently, non-coding RNAs (miRNAs and long non-coding RNAs) emerge as a crucial regulator of MCs development and play an important role in allergic diseases and bronchial asthma. Loss of dicer function in mouse MC progenitor cells results in profound disruption of tissue MC compartments, suggesting a critical role for miRNAs in MC differentiation, growth, and migration (49). miR-221 has been shown to regulate the cell cycle and cytoskeleton development process in MCs, while in response to stimulation through IgE-antigen complexes, miR-221 shows MCs’ specific effect and leads to degranulation and cytokine release (50). Higher expression (~3-fold) of miR-221 has also been reported in a murine lung asthma model compared to control mice (51). A potential increase in expression of miR-221 was also noted in P815 mouse MCs after lipopolysaccharide stimulation (51). In addition, the authors further demonstrated that miR-221 overexpression increases total cells and eosinophil numbers in the murine asthma model and stimulates IL-4 secretion in MCs through PTEN, p38, and NF-κB pathways. Upregulation of miR-221 and miR-485-3p has been reported in the peripheral blood mononuclear cells of pediatric asthmatic patients (*n* = 6), compared to control-group children. Similarly, upregulation (~3-fold) of miR-221 and miR-485-3p has been noted in the lungs of OVA-induced asthmatic mouse model and resulting into decreased levels of their target gene, Spred-2 (52). In a separate study, it was shown that inhibition of miRNA-221 suppresses the airway inflammation in asthmatic mouse model (53). These findings suggest that miR-221 might play an important role in the onset and development of asthma. Similarly, miR-21 also been shown to be upregulated in the multiple asthmatic mouse models induced by house dust mite, OVA, or *Aspergillus fumigatus* (54). miR-21 also suppresses TLR2 signaling pathways in inflammatory lung mouse model (55). In addition, miR-21 is also upregulated in IL-13-treated primary normal human airway epithelial cells. There are only few dedicated studies investigating the role of long non-coding RNA (lncRNA) in asthma. In this context, Zhang et al. have detected around 3.5-fold higher expression of BCYRN1 lncRNA in the ASM cells of the asthmatic rat model, compared to control group. In addition, BCYRN1 level increases in ASM cells in the presence of PDGF. Furthermore, BCYRN1 promotes the proliferation and migration of ASM cells *via* upregulating the expression of transient receptor potential 1 (TRPC1) protein through increasing the stability of TRPC1 (56). The overexpression of TRPC1 reversed the function of si-BCYRN1 in decreasing viability/proliferation and migration of ASM cells. Recently, plasmacytoma variant translocation (PVT1), another lncRNA, has been shown to decrease in ASM of patients with corticosteroid-sensitive non-severe asthma. However, its levels are increased in the patients with corticosteroid-insensitive severe asthmatic patients (57). Furthermore, the authors have shown that PVT1 regulates both IL-6 release and TGF-β-induced proliferative response in ASM cell in severe asthmatic patients. Similarly, growth arrest specific 5 lncRNA has been demonstrated to correlate with the corticosteroid response in severe asthmatic patients (58).

Several miRNAs have been demonstrated to target the components of the S1P signaling pathway (Table 1). For example, miR-124 and miR-506 target SphK1 (55, 59, 60), miR-130a-3p and miR-613 target SphK2 (61, 62), and miR-125b-1-3p, miR-133b, and miR-363 target S1PR1 (63–65), whereas miR-125b (66) binds to the S1P lyase mRNA transcript. However, the role of above miRNAs has not been elucidated in MC functions and need to be further investigated to explore the interaction between miRNA and S1P signaling pathway.

**Table 1:** **Components of sphingosine-1-phosphate (S1P) signaling pathways targeted by miRNAs**.

S. no.	miRNAs	Components of S1P signaling pathways affected	Reference
1	miR-124	Sphingosine kinase 1 (*SPHK1*)	(55)
2	miR-506	*SPHK1*	(59, 60)
3	miR-130a-3p	Sphingosine-1-phosphate receptor 2 (*S1PR2*)	(61)
4	miR-613	Sphingosine kinase 2 *(SPHK2)*	(62)
5	miR-125b-1-3p	Sphingosine-1-phosphate receptor 1 (*S1PR1*)	(63)
6	miR-133b	*S1PR1*	(64)
7	miR-363	*S1PR1*	(65)
8	miR-125b	Sphingosine-1-phosphate lyase 1 (*SGPL1*)	(66)

## Concluding Remarks

Sphingosine-1-phosphate is a dual regulator of the systemic allergic responses. It is an essential signaling molecule generated upon IgE-FcεRI crosslinking and enhances the activation of MCs in allergic diseases such as asthma. On the other hand, S1P signaling through its receptors is required for recovery from anaphylactic shock. The role of non-coding RNAs is well explored in cancer; however, their role in allergy and inflammation is still in infancy, and their role needs to be explored as a potential therapeutic target and biomarker for asthma.

## Author Contributions

RS, AK, MJ, SG, and AJ designed the manuscript; were involved in drafting/revising the manuscript; and read and approved the final manuscript. RS, AK, and AJ wrote the first draft.

## Conflict of Interest Statement

The authors declare that the research was conducted in the absence of any commercial or financial relationships that could be construed as a potential conflict of interest.
